# Newly-Discovered Neural Features Expand the Pathobiological Knowledge of Blastic Plasmacytoid Dendritic Cell Neoplasm

**DOI:** 10.3390/cancers13184680

**Published:** 2021-09-18

**Authors:** Maria Rosaria Sapienza, Giuseppe Benvenuto, Manuela Ferracin, Saveria Mazzara, Fabio Fuligni, Claudio Tripodo, Beatrice Belmonte, Daniele Fanoni, Federica Melle, Giovanna Motta, Valentina Tabanelli, Jessica Consiglio, Vincenzo Mazzara, Marcello Del Corvo, Stefano Fiori, Alessandro Pileri, Gaetano Ivan Dellino, Lorenzo Cerroni, Fabio Facchetti, Emilio Berti, Elena Sabattini, Marco Paulli, Carlo Maria Croce, Stefano A. Pileri

**Affiliations:** 1 Division of Haematopathology, IEO, European Institute of Oncology IRCCS, 20141 Milan, Italy; saveria.mazzara@ieo.it (S.M.); Federica.Melle@ieo.it (F.M.); Giovanna.Motta@ieo.it (G.M.); valentina.tabanelli@ieo.it (V.T.); v.mazzara@campus.unimib.it (V.M.); marcello.delcorvo@ieo.it (M.D.C.); stefano.fiori@ieo.it (S.F.); 2 Department of Biology, University of Padua, 35122 Padua, Italy; giuseppe.benvenuto@bmr-genomics.it; 3 Department of Experimental, Diagnostic and Specialty Medicine, University of Bologna, 40138 Bologna, Italy; manuela.ferracin@unibo.it (M.F.); alessandro.pileri2@unibo.it (A.P.); 4 Department of Genetics and Genome Biology, The Hospital for Sick Children, Toronto, ON M5G 0A4, Canada; fabio.fuligni@sickkids.ca; 5 Tumor Immunology Unit, Human Pathology Section, Department of Health Science, Palermo University School of Medicine, 90134 Palermo, Italy; claudio.tripodo@unipa.it (C.T.); beatrice.belmonte@unipa.it (B.B.); 6 Department of Pathophysiology and Transplantation, University of Milan, 20122 Milan, Italy; daniele.fanoni@unimi.it (D.F.); emilio.berti@unimi.it (E.B.); 7 Department of Molecular Virology, Immunology and Medical Genetics, Ohio State University, Columbus, OH 43210, USA; j.consiglio3@gmail.com (J.C.); carlo.croce@osumc.edu (C.M.C.); 8 Department of Experimental Oncology, European Institute of Oncology, 20141 Milan, Italy; gaetano.dellino@ieo.it; 9 Die Dermatopathologie der Universitätsklinik für Dermatologie und Venerologie, LKH-Univ. Klinikum Graz, 8036 Graz, Austria; lorenzo.cerroni@medunigraz.at; 10 Pathology Section, Department of Molecular and Translational Medicine, University of Brescia, 25123 Brescia, Italy; fabio.facchetti@unibs.it; 11 Department of Dermatology, Fondazione IRCCS Ca’ Granda-Ospedale Maggiore Policlinic and Milan University, 20122 Milan, Italy; 12 Haematopathology Unit, IRCCS Azienda Ospedaliero-Universitaria di Bologna, 40138 Bologna, Italy; elena.sabattini@aosp.bo.it; 13 Unit of Anatomic Pathology, Department of Molecular Medicine, University of Pavia and Fondazione IRCCS San Matteo Polyclinic, 27100 Pavia, Italy; marco.paulli@unipv.it

**Keywords:** BPDCN, miRNA, neurogenesis, network, sequencing

## Abstract

**Simple Summary:**

For the first time, neuronal features are described in blastic plasmacytoid dendritic cell neoplasm (BPDCN) by a complex array of molecular techniques, including microRNA and gene expression profiling, RNA and Chromatin immunoprecipitation sequencing, and immunohistochemistry. The discovery of unexpected neural features in BPDCN may change our vision of this disease, leading to the designing of a new BPDCN cell model and to re-thinking the relations occurring between BPDCN and nervous system. The observed findings contribute to explaining the extreme tumor aggressiveness and also to propose novel therapeutic targets. In view of this, the identification, in this work of new potential neural metastatic inducers might open the way to therapeutic approaches for BPDCN patients based on the use of anti-neurogenic agents.

**Abstract:**

Blastic plasmacytoid dendritic cell neoplasm (BPDCN) is a rare and highly aggressive hematologic malignancy originating from plasmacytoid dendritic cells (pDCs). The microRNA expression profile of BPDCN was compared to that of normal pDCs and the impact of miRNA dysregulation on the BPDCN transcriptional program was assessed. MiRNA and gene expression profiling data were integrated to obtain the BPDCN miRNA-regulatory network. The biological process mainly dysregulated by this network was predicted to be neurogenesis, a phenomenon raising growing interest in solid tumors. Neurogenesis was explored in BPDCN by querying different molecular sources (RNA sequencing, Chromatin immunoprecipitation-sequencing, and immunohistochemistry). It was shown that BPDCN cells upregulated neural mitogen genes possibly critical for tumor dissemination, expressed neuronal progenitor markers involved in cell migration, exchanged acetylcholine neurotransmitter, and overexpressed multiple neural receptors that may stimulate tumor proliferation, migration and cross-talk with the nervous system. Most neural genes upregulated in BPDCN are currently investigated as therapeutic targets.

## 1. Introduction

Blastic plasmacytoid dendritic cell neoplasm (BPDCN) is a rare hematologic malignancy, variably affecting the skin, bone marrow, peripheral blood, lymph nodes, and the central nervous system (CNS) [1,2]. In the past, it was mistakenly named blastic NK/T-cell lymphoma or agranular CD4+/CD56+ hematodermic neoplasm/tumor [3]. In 2008, it was classified as a neoplasm deriving from precursors of plasmacytoid dendritic cells (pDCs), also known as professional type I interferon producing cells, and provisionally included among acute myeloid leukemias (AMLs) [4]. In 2017, the Revised 4th Edition of the WHO Classification of Haematopoietic and Lymphoid Tumours recognized BPDCN as a unique entity distinct from AML [1].

BPDCN represents less than 1% of hematologic malignancies [1]. It often occurs in males in the sixth decade of life and is characterized by a dismal prognosis (median overall survival of 12–14 months) [1]. In the absence of a standard therapy, BPDCN patients are variably treated with schedules applied to acute myeloid leukemia (AML), acute lymphoblastic leukemia (ALL), or aggressive lymphoma, followed by stem cell transplantation upon first remission. However, they frequently relapse and die [5]. Of note, about 30% of relapses occur at the central nervous system (CNS) level [2].

Over the last few years, gene expression profiling, cytogenetic and next generation sequencing studies have provided relevant information as to the molecular characteristics of the tumor. BPDCN presented a complex karyotype, frequent deletions of tumor-suppressor genes (including *CDKN2A/1B*, *TP53*, *RB1*, *TET2*, and *PTEN*) [6], deregulation of NF-kB and cholesterol pathways [7,8], recurrent mutations affecting either the DNA methylation or chromatin-remodeling pathways [9,10] and transcriptional dependency on bromodomain proteins [11]. These findings have contributed to the proposal of new therapeutic approaches that are providing promising results, although concerns still remain regarding their safety [12,13]. Thus, there is still the need to understand better the mechanisms sustaining tumor aggressiveness and to search for further therapeutic options. In this respect, only one study focused on microRNA expression in BPDCN but as compared with myeloid sarcoma [14]. MicroRNAs (miRNAs) are small non-coding regulators playing a central role in the control of gene translation and modulation of the biological pathways in various cancer types [15]. Their investigation might shed new light on the pathobiology of the tumor.

In this study, the microRNA profile of BPDCN was investigated by the NanoString array technology, aiming to (1) determine its possible contribution to the transcriptional program and (2) uncover new molecular features sustaining neoplastic growth and progression.

## 2. Materials and Methods

### 2.1. Case Collection and Study Design

Nineteen BPDCN biopsies obtained at the time of diagnosis and 4 pDCs samples represented the discovery set. The 19 BPDCN tumor samples included 4 lymph-node and 15 skin biopsies, 13 of which were paraffin-embedded (FFPE) and 6 frozen tissues, as detailed in Table S1. All the 19 BPDCN cases had been reviewed by a panel of experienced hemato-pathologists (VT, SF, ES and SAP), classified by immunohistochemistry evaluation, according to the criteria of the WHO Classification of Tumours of Haematopoietic and Lymphoid Tissues [1]. Samples were included in the study if showing at least 80% of neoplastic cells and good RNA preservation.

The patients’ age and sex, type of examined sample, clinical information available concerning the dissemination of disease, the type of therapy employed and follow-up are reported in Table S1.

The panel of antibodies used for the diagnosis included the following markers: CD4 (Leica 4B12), CD56 (Dako 123C3), TdT (Dako EP266), CD123 (BD Pharmingen 7G3), CD303/BDCA2 (EuroBio 124B3.3), TCL1 (Invitrogen 1–21), CD68/PGM1 [16], MPO (Dako Polyclonal), CD34 (Leica Qbend10), CD3 (AbCam SP162), CD20 (Dako L26), and CD30 (Dako ber-H2). The main immunohistochemical characteristics of the BPDCN patients are shown in Table S2.

Informed consent was obtained from each patient in accordance with the guidelines of the Institutional Review Board of the Department of Experimental, Diagnostic, and Specialty Medicine of the University of Bologna and the Declaration of Helsinki.

The 4 pDCs samples were isolated from the peripheral blood of healthy donors as previously described [7]. The microRNA profile of the discovery set was obtained by the NanoString human miRNA assay technology. All samples of the discovery set were also provided with gene expression data (GSE62014) [7] that were used to assess the possible impact of miRNAs on the regulation of the transcription.

The molecular findings derived from the microRNA profiling of the discovery set were validated by quantitative reverse transcription PCR (RT-qPCR) and by immunohistochemistry on an independent validation set of 15 FFPE BPDCN collected at diagnosis.

The molecular results derived from the discovery set were also validated and further explored in silico by using: two RNA sequencing validation sets [10,11] and one H3K27me3/ac chromatin immunoprecipitation sequencing (ChIP) validation set [10]. The study design was illustrated in Figure S1.

#### 2.1.1. RNA Sequencing Validation Sets

The molecular results of the discovery set were validated in silico in two distinct RNA sequencing sets. The former set was composed of 9 BPDCNs and 4 pDC samples, whose molecular data had been previously published by our group [10] (GSE164939). Since six of these cases were included in the discovery set of the present study, this set was used for both technical and experimental validation.

The latter RNA sequencing validation set was composed of 6 BPDCN and 6 pDC samples, previously reported by Ceribelli et al. (GSE84471) [11].

These two RNA sequencing validation sets were analyzed by differential analysis and Gene Set Enrichment Analysis (GSEA) as reported in Supplementary File.

The expression of selected genes was also evaluated in 12 BPDCN, 65 acute myeloid leukemia (AML) and 35 T-Cell Lymphoblastic Leukemia (T-ALL) cases, whose gene expression profiling data were available in silico (GSE89565) [8].

#### 2.1.2. H3K27me3/ac ChIP Sequencing Independent Validation Set

To extend the molecular investigation at the epigenetic level, the H3K27me3/Ac ChIP sequencing data of 2 BPDCN cases were used, which had been included in a previous publication [10].

### 2.2. MicroRNA Expression Profiling Discovery Set

RNA was extracted from discovery set samples (19 BPDCNs and 4 normal pDCs), loaded on the multiplexed NanoString nCounter Human miRNA expression assays 1.0 and then processed and normalized according to manufacturer’s instructions (NanoString Technologies, Inc., Seattle, WA, USA). Hierarchical clustering was performed with Euclidean distance and average-linkage method. Differential expression analysis between BPDCNs and pDCs was carried out by the two-class unpaired test in Samr R package. MicroRNAs with a logFC > |1.5| and FDR < 0.05, were recognized as differentially expressed between BPDCN tumor and pDC control samples.

### 2.3. MicroRNA Expression Quantification by Quantitative Reverse Transcription PCR (RT-qPCR)

Randomly selected miRNAs were evaluated by quantitative reverse transcription PCR (RT-qPCR) as detailed in the Supplementary File.

### 2.4. BPDCN Gene Expression Profiling Analysis Discovery Set

BPDCN raw gene expression signals were downloaded from GEO database (GSE62014) [7] and analyzed as detailed in the Supplementary File.

### 2.5. MicroRNA Network Analysis

MicroRNA and gene expression data were analyzed by the Micrographite pipeline in order to identify relevant miRNA-gene interactions by pathway analysis [17]. Differentially expressed miRNAs (logFC > |1.0|, FDR < 0.05), that significantly correlated or anticorrelated (Spearman’s coefficient > |0.6|) with differentially expressed target genes (logFC > |2.5|, FDR < 0.05) were considered for network analysis. Since a significant portion of miRNA-gene interactions were not considered by the Micrographite pipeline, the TargetScanHuman database was also used [18]. MiRNA target genes experimentally validated, according to TargetScanHuman database, were added to the network if differentially expressed in the BPDCN vs. pDC discovery set samples (logFC > |2.5| and FDR < 0.05). Cytoscape 3.8.2 was applied to visualize the integrated regulatory network [19].

The network genes were then interrogated by Gene Ontology (GO) as detailed in Supplementary File.

### 2.6. Immunohistochemistry Validation Set

Fifteen BPDCN cases of the Immunohistochemistry validation set underwent immunohistochemistry (IHC) as reported in Supplementary File. The clinical characteristics of these cases are summarized in Table S3.

## 3. Results

### 3.1. BPDCN Cases Display a Set of 51 miRNAs Differentially Expressed from Normal pDCs

Nineteen BPDCN cases and 4 pDCs samples representing the discovery set were analyzed by the NanoString nCounter human miRNA assay (NanoString Technologies, Seattle, WA, USA), according to the study design (Figure S1). Clinical patients’ characteristics are reported in Table S1.

**Figure 1:**
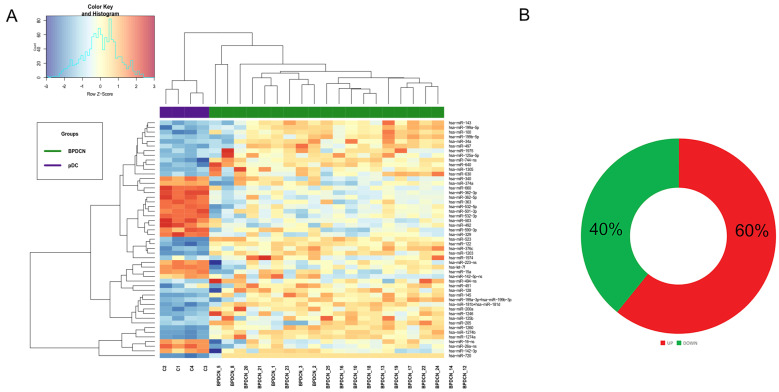
MicroRNA expression profiling of in blastic plasmacytoid dendritic cell neoplasm (BPDCN). (**A**) Heat map of genes differentially expressed in BPDCNs vs. plasmacytoid dendritic cells (pDCs), according to differential expression analysis. Tumor BPDCN samples are reported in green and pDC control samples in purple; (**B**) MiRNAs upregulated and downregulated are graphically represented by a donut chart.

Hierarchical Clustering demonstrated that BPDCNs and pDCs grouped separately, according to different miRNA expression values (Figure 1A). Specifically, 51 miRNAs were significantly deregulated in tumor samples versus normal pDCs: 31 were upregulated and 20 downregulated (FDR < 0.05 and Log FC > |1.5|) (Figure 1B, Table S4).

Randomly selected miRNAs were validated by qRT-PCR (Figure S2).

Next, we interrogated the 51 BPDCN deregulated miRNAs by miRNet bioinformatic Tool [20] and miRCancer database [21] and found that 9 miRNAs were in common with acute myeloid leukemia (AML), in line with their molecular relatedness [7]. In particular, BPDCN turned out to share with AML the upregulation of miR-181a and miR-125b, responsible for RAS signaling dysregulation in AML [22] and leukemia development an in vivo mouse model, respectively [23]. In addition, both BPDCN and AML downregulated the tumor suppressor miR-223, with possible loss of its pro-apoptotic function [24] (Table S5).

### 3.2. BPDCN miRNA-mRNA Regulatory Network May Primarily Affect Neurogenesis

All samples of the discovery set were also provided with gene expression data (GSE62014) [7] that we used to assess the possible impact of miRNAs on the regulation of the transcriptional process.

**Figure 2:**
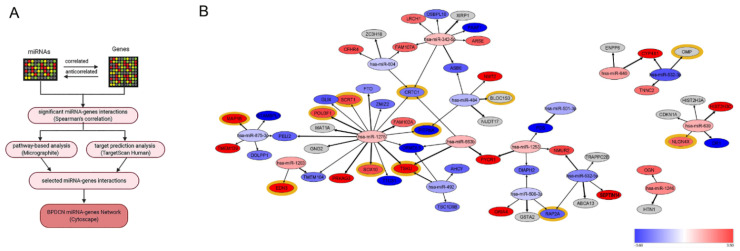
MicroRNA network analysis of BPDCN. (**A**) Flow chart of network analysis. MiRNA-genes interactions were analyzed by pathway analyses using Micrographite, so that only miRNA-target genes involved in the most modulated pathways were selected. In parallel, miRNA-gene interactions were enriched by target prediction analysis, using TargetScan. As a conclusive step, the selected miRNA-gene interactions were represented by the Cytoscape tool; (**B**) BPDCN miRNA network model visualized by Cytoscape 3.8.2. Genes and miRNAs were reported as circles. Node colors represented the expression fold-change of miRNAs and genes in the BPDCNs vs. pDCs. The thickness of edges was proportional to the strength of interaction. Highlighted in yellow the network genes predicted to be involved in neurogenesis (GO:0022008).

As reported in Figure 2A, the miRNA and gene expression profiles were integrated by performing a multistep network analysis. Firstly, only miRNA–targets with significant anti-correlated and correlated interactions were selected. Next—given that miRNA expression is not always anti-correlated with target genes but more often its regulatory function may depend on the pathway, which is involved in [25]—the Micrographite pipeline was applied to identify relevant miRNA-gene interactions by pathway-analysis [17]. The resulting miRNA-gene interactions were validated and integrated by target prediction analysis. Finally, using this multistep approach based on the combination of experimental and predictive results, we identified a robust non-redundant BPDCN network composed of 16 miRNAs and 57 genes (Figure 2B, Table S6).

**Figure 3:**
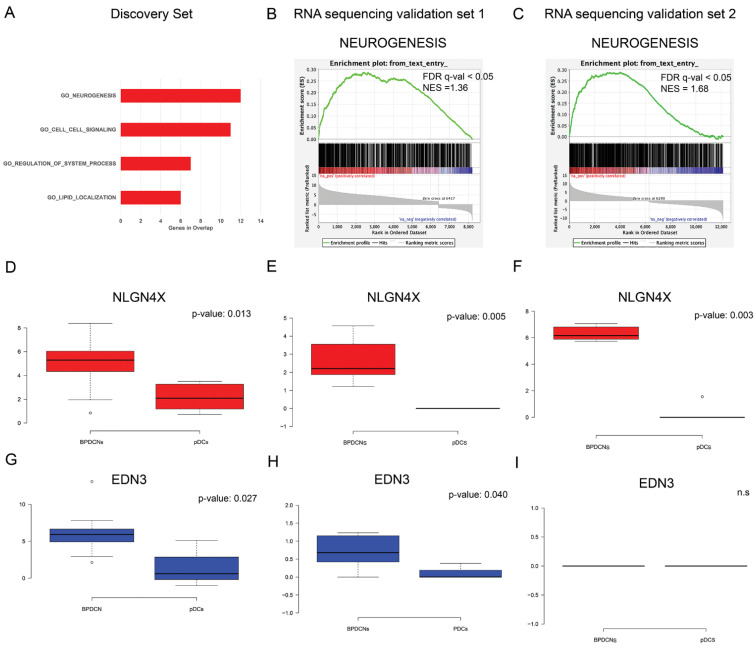
MiRNA network functional analysis. (**A**) The top four biological processes emerged, as significantly enriched in 57 network-genes by gene ontology (GO) analysis, are reported from the top to the bottom, from the most enriched to the lowest; (**B**,**C**) Pre-ranked Gene set enrichment analysis (GSEA) indicated that GO_neurogenesis gene set was significantly enriched in the RNA sequencing transcriptome of BPDCN compared with pDC samples in RNA sequencing validation set 1 and 2, respectively. Normalized enrichment scores (NES) and false discovery rate (FDR) *q*-values are given for the gene set. (**D**–**F**) Box plots showed in red the relative mRNA expression of *NLGN4X* in discovery set in RNA sequencing validation set 1 and 2, respectively. *NLGN4X* was significantly overexpressed in BPDCNs vs. pDCs in all the analyzed sets. (**G**–**I**) Box plots showed in blue the relative mRNA expression of *EDN3* in discovery set and in RNA sequencing validation set 1 and 2, respectively. *EDN3* was significantly overexpressed in the discovery set and RNA sequencing validation set. Box plots were created by webtool BoxPlotR. Center lines show the medians; box limits indicate the 25th and 75th percentiles as determined by R software; whiskers extend 1.5 times the interquartile range from the 25th and 75th percentiles, outliers are represented by dots. Statistical analysis was performed using Kruskal–Wallis rank sum test for multiple independent samples.

To understand the biological implications of the identified miRNA regulatory network, the 57 network-genes were interrogated by functional prediction analysis of biological processes. Gene set enrichment analysis (GSEA) [26], based on Gene Ontology terms (GO), revealed that neurogenesis was the biological process most significantly influenced by miRNA dysregulation, followed by cell-cell signaling, regulation of system process, and lipid localization (FDR *q*-value < 0.05) (Figure 3A). Specifically, 12 out of 57 BPDCN network-genes were included in the GO_neurogenesis gene list (GO:0022008) (Figure 2B). In view of these findings, we focused our interest on neurogenesis.

First of all, independent BPDCN series were investigated to see whether they also showed significant neurogenesis process upregulation. A pre-ranked GSEA of the GO_neurogenesis gene list (GO:0022008) was carried out in two in silico RNA sequencing validation sets: both confirmed the significant enrichment of neurogenic process, with a FDR *q*-value < 0.05 and a Normalized Enrichment Score (NES) ≥ 1.36 and ≥1.68, respectively (Figure 3B,C). Secondly, at the light of literature findings, among the network-genes related to neurogenesis (Figure 2B), the attention was focused on Neuroligin 4 X-linked (*NLGN4X*) and Endothelin-3 (*EDN3*), never reported before in BPDCN but already known to correlate with tumor aggressiveness [27,28,29]. *NLGN4X* was aberrantly overexpressed in BPDCNs vs. normal pDCs in the discovery set and both RNA sequencing validation sets (Figure 3D–F, *p*-value ≤ 0.05). *EDN3* was significantly overexpressed in BPDCNs vs. normal pDCs in the discovery set and validation set 1, but not in validation set 2 (Figure 3G–I, *p*-value ≤ 0.05). Given the promising role of NLGN4X and EDN3 as therapeutic targets and prognostic factors in cancer patients [27,28,29], their predictive value in the discovery set was evaluated. But their expression level was not significantly associated with the BPDCN clinical data at our disposal. To evaluate the specificity of *EDN3* and *NLGN4X* to BPDCN, we analyzed their expression in 12 BPDCN vs. 35 T-Cell Acute Lymphoblastic Leukemia (T-ALL) and vs. 65 AML cases, available in silico (GSE89565). *EDN3* was significantly upregulated in AML vs. BPDCN and *NLGN4X* was significantly upregulated in BPDCN vs. AML (Figure S3).

### 3.3. Unexpected Neuronal Features in BPDCN

To explore neurogenesis in BPDCN, the immunohistochemical expression of well-known neural markers was assessed in an independent cohort of 15 BPDCN skin biopsies (detailed in Table S2).

Recent studies have shown that neural cells comprised within tumor microenvironment may promote tumoral growth. In prostatic [30], gastric [31] and breast cancer [32], tumor cells recruit newly formed nerves and neural cells in the surrounding stroma, in order to sustain tumor progression and metastasis.

Thus, antibodies against neurofilament light chain (NF-L) and neurofilament heavy chain (NF-H) were applied to detect newly formed and mature nerve fibers, respectively, in BPDCN. All 15 cases resulted negative for both markers: no neural fibers were running in the tumor microenvironment (Figure S4).

**Figure 4:**
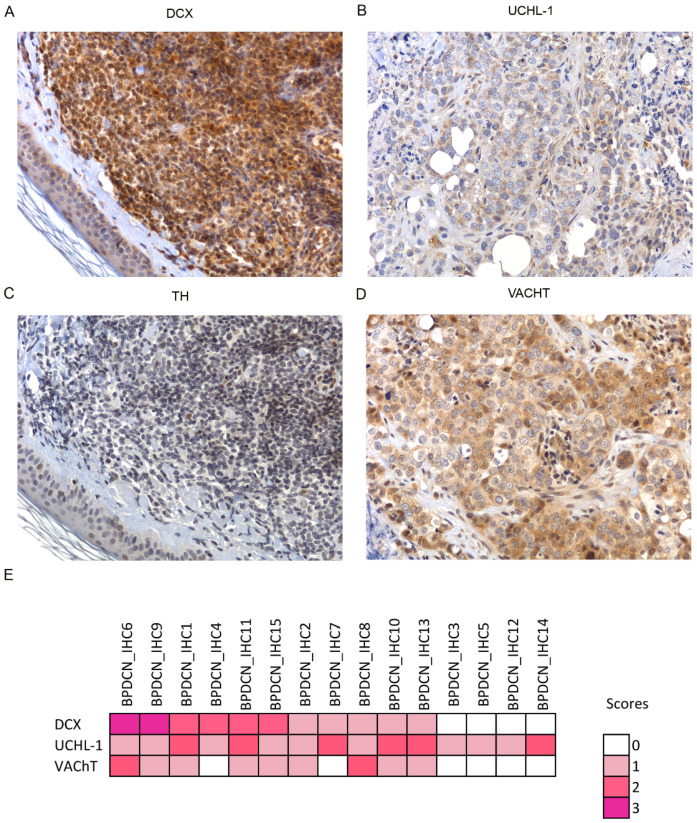
Immunostaining for neural markers in BPDCN. Immunohistochemistry assay shows the positivity for DCX (**A**) UCHL-1 (**B**) and VAChT (**D**), in the BPDCN tumor cells of selected BPDCN samples. (**C**) All the BPDCN samples, except one, were negative for TUBB3 as illustrated. (**E**), Heat map shows the immunophenotypic findings in the 15 BPDCN samples of the immunohistochemistry validation set. The color intensity corresponds to the ordinal score. Microphotographs were collected using a Zeiss Axiocam 503 Color digital camera using the Zen 2.0 imaging software. Original magnifications × 20.

Next, to evaluate the presence of neural cells in BPDCN, antibodies anti—doublecortin (DCX), and Ubiquitin C-terminal hydrolase 1 (UCHL-1) were used. DCX is a neural progenitor marker involved in adult neurogenesis of CNS. Recent studies demonstrated that DCX may be responsible for cancer metastasis and uncontrolled migration of cancer cells [30,33]. UCHL-1 is a neural marker that regulates neural progenitor cell differentiation, thereby enhancing neurogenesis in the embryonic brain [34]. No neural cells were detected. Instead, the tumor cells themselves turned out to express neural markers; 11/15 cases stained for DCX (6/11 at high score level) and all cases were positive for UCHL-1 (6/15 cases at high level) (Figure 4A,B,E).

In addition to DCX and UCHL-1, the positivity of BPDCN samples for β- III tubulin (TUBB3) was tested. TUBB3 is a protein expressed by neurons as well as neural crest-derived cells, like melanocytes [35]. While the residual melanocytes and/or endothelial cells present in each sample were positive for TUBB3, BPDCN tumor cells turned out negative in all instances but one (Figure S4).

Having observed that BPDCN tumor cells retained some neural properties, the question was raised as to whether BPDCN cells might mediate neural signals by neurotransmitter exchange. The most relevant neurotransmitters of the peripheral nervous system are acetylcholine and catecholamines. The expression of tyrosine hydroxylase (TH) and vesicular acetylcholine transporter (VAChT) proteins was investigated by IHC. The enzyme TH is essential for the catecholamine biosynthesis and its expression level is proportional to catecholamine production [36], while, VAChT protein mediates the intracellular storage and release of Acetylcholine (ACh), thus representing its rate-limiting factor [37]. All BPDCN cases except one resulted TH-negative but more than a half (9/15, 60%) stained for VAChT and 2/9 cases at high level (Figure 4C–E).

In summary, the catecholamine biosynthesis appeared globally shut down, while the ACh exchange was presumably active in more than half of the cases.

### 3.4. A New BPDCN Cell Model

To acquire a more comprehensive understanding of neural modulation of BPDCN cells, microRNA profiling results were matched with the in silico data of RNA sequencing validation sets and H3K27me3/ac chromatin-immunoprecipitation sequencing (ChIP-seq) [10]. According to RNA sequencing differential analysis of BPDCNs vs. pDCs, we found upregulated: the acetylcholinesterase (ACHE) and butyrylcholinesterase (BCHE) genes, involved in the modulation of cholinergic signaling, and also the ACh receptors genes, both the nicotinic (e.g., CHRNA3/4/5) and the muscarinic (e.g., CHRM1/2)] receptors genes, that mediates the transmission of Ach and the activation of the signal transduction cascade, respectively [38]. These findings provided further evidence of the molecular activation of cholinergic signaling in BPDCN.

Moreover, additional neural related genes were found upregulated: the nerve growth factor (NGF), its receptor (NGFR), the Neurotrophic Tyrosine Kinase Receptor (NTRK1/2/3), and the gamma-aminobutyric acid (GABA) receptor subunit genes (e.g., GABRA2/3/4).

Overall, thirty-five neural-related genes were significantly upregulated in RNA sequencing data of BPDCN vs. pDCs (padj ≤ 0.05, fold change >1.0), Table S3.

The epigenetic status of these 35 genes was assessed by ChIP-seq. Specifically, ChIP-seq in silico data were used to analyze the BPDCN genome-wide distribution of H3K27 acetylation (H3K27ac), an epigenetic inducer of gene transcription [10]. It was found that Neurotrophic Tyrosine Kinase Receptor gene (NTRK1) was acetylated in both BPDCN cases and Cholinergic Receptor Nicotinic Beta 1 (CHRNB1) and GABA Type A Receptor-Associated Protein (GABARAP) genes in one case, leading to hypothesize a possible role of H3K27ac in neural signaling induction.

Finally, as to the role of microRNAs in the post-transcriptional modulation of cholinergic signaling, miR-125b and miR-18 were found upregulated: they are known to interfere with the cholinergic system and thus called “CholinomiRs” [39].

**Figure 5:**
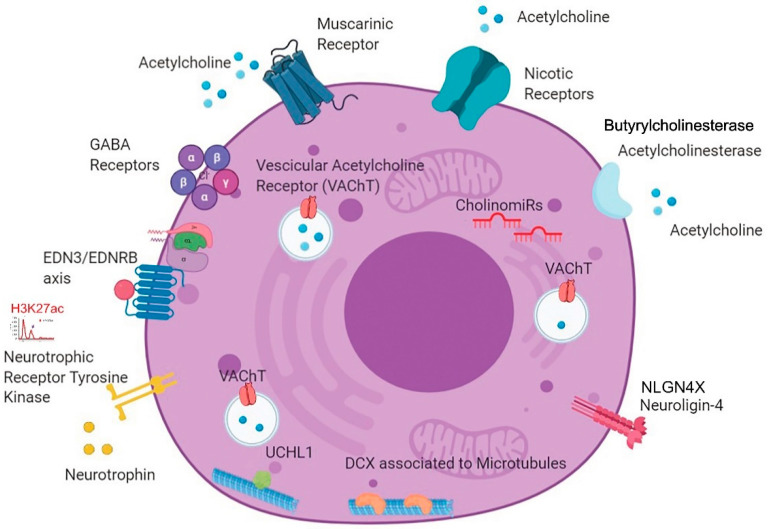
New BPDCN cell model. The cartoon summarizes all the neural features detected in BPDCN tumor cells by IHC (positivity for DCX, UCHL-1 and VAChT), RNA sequencing (overexpression of muscarinic and nicotinic receptors for acetylcholine, butyrylcholinesterase and acetylcholinesterase genes, GABA receptors, neurotrophins and neurotrophic receptor tyrosine kinase genes), miRNA network analysis (EDN3 and NLGN4X), miRNA expression profiling (deregulation of CholinomiRs,) and chromatin immunoprecipitation sequencing (H3K27ac). (Created with BioRender.com, accessed on 21 August 2020).

In summary, by interrogating different molecular sources, unprecedented neuronal features of BPDCN were discovered, which are graphically synthesized in Figure 5.

## 4. Discussion

In this study, we discovered in BPDCN new unexpected molecular features related to neurogenesis, possibly involved in tumor cell dissemination.

The primary aim of this study was to evaluate the expression level of miRNAs in BPDCN compared to normal pDC discovery set samples. First of all, 51 miRNAs significantly deregulated in BPDCN tumor samples were identified—some of them in common with AML, further supporting the molecular relatedness of the two diseases [7]. Secondly, to evaluate the impact of miRNA dysregulation on BPDCN transcriptional program, miRNA and gene expression data of discovery set samples were integrated and interrogated by a multi-step network analysis. We finally identified a BPDCN miRNA regulatory network (including 16 miRNAs and 57 genes) whose main function was predicted to be the modulation of neurogenesis.

The neurogenesis, seen as the orchestration of nerve fibers and neuron outgrowth, is a biological process that is causing increased interest in solid tumors: in gastric and breast carcinomas, cancer stem cells have shown to produce neurons that stimulate tumor neurogenesis and tumor growth [31,32], while in prostatic tumors, neural progenitors from the central nervous system (CNS) may migrate to the prostate, initiate tumor innervation and promote metastases [30]. The neurogenesis has been only marginally investigated in hematological malignancies and never in BPDCN. To our knowledge, only in 2005, Dijk-man et al. mentioned the upregulation of a set of neuronal-related genes in BPDCN versus AML [40]. Later on, Sapienza et al. reported that DNA mutations significantly affected gamma-aminobutyric acid (GABA) neural signaling [10]. However, none of these preliminary observations were subsequently further investigated.

In the present work, starting from miRNA network molecular findings, we decided to explore and better characterize the neurogenesis in BPDCN.

First, we focused on the neuroligin 4 X (*NLGN4X*) and Endothelin 3 (*EDN3*) genes, two BPDCN network neural genes, aberrantly upregulated in BPDCN samples (Figure 3) and already described in the literature as possibly involved in tumor progression [27,28,29]. *NLGN4X* encodes for a brain-specific cell adhesion molecule, abundantly expressed in the cerebral cortex. It is also expressed in breast cancer, at higher levels in metastatic tissues, where it represents a novel potential biomarker and an attractive therapeutic target [27]. *EDN3* has been found upregulated in glioblastoma stem cells, melanoma, and breast cancer and similarly to *NLGN4X*, promotes tumor proliferation and invasiveness [28,29]. As its inhibition may induce cell arrest, *EDN3* gene has been suggested as a new therapeutic option for glioblastoma, melanoma and more in general for cancer patients overexpressing it [28,29]. Their expression was further tested in two external BPDCN RNA sequencing validation sets: *NLGN4X* was upregulated in both sets, while *EDN3* was upregulated in only one. Unfortunately, the number of patients with available clinical data (discovery set = 19) was statistically too low to evaluate the association of *NLGN4X* and *EDN3* expression level with tumor dissemination and patient survival.

Secondly, to explore the phenomenon of neurogenesis in BPDCN, we decided to evaluate the presence of nerve fibers and neurons in the tumor microenvironment. Thus, an independent set of 15 BPDCN cutaneous biopsies was interrogated by specific immunohistochemical neural markers, capable of detecting nerves (NF-L and NF-H) and neural progenitors (DCX and UCHL-1).

The BPDCN microenvironment resulted negative for neural markers but, surprisingly, the tumor cells themselves were positive for the 2 progenitor neural markers: DCX and UCHL-1, in 11/15 (73.3%) and 15/15 (100%) cases, respectively (Figure 4). DCX is a microtubule-associated protein, involved in cytoskeletal remodeling and neuroblasts migration. It is also expressed in a variety of cancers where it promotes tumor cell mobility and metastasis [33]. The second neural marker, UCHL-1 (alias PGP9.5), is a unique brain-specific deubiquitinating enzyme upregulated also in neuroblastoma, acute lymphoblastic leukemia, non-small cell lung cancer, and renal cell carcinoma [34,41]. Of clinical interest, its inhibition might reduce cell invasiveness [38].

Since the expression *DCX* and *UCHL-1* may increase the ability of BPDCN tumor cells to disseminate to distant organs, globally worsening patient outcome, both genes, as already suggested for *NLGN4X* and *EDN3*, may be regarded as possible therapeutic targets to arrest BPDCN dissemination.

To test whether this neuronal-like immunophenotype may reflect the acquisition of new neuronal functions, and specifically, the ability of BPDCN cells to transmit neuronal signals, the intracellular expression of catecholamines and acetylcholine was also investigated by immunohistochemistry (IHC). Catecholamines and acetylcholine are the two main neurotransmitters of the peripheral nervous system, capable of stimulating or inhibiting neuronal activity. According to IHC results, all BPDCN cases were negative for the tyrosine hydroxylase enzyme responsible for dopamine biosynthesis [33], while more than half BPDCN cases (9/15) were positive for the vesicular transporter of acetylcholine that is the mediator of acetylcholine storage/release and the rate-limiting factor of its cellular neurotransmission (Figure 4) [37]. Therefore, BPDCN cells showed they were able to transmit acetylcholine-based neural signals.

Acetylcholine (ACh) is a key mediator in the central and peripheral nervous systems playing an important role in learning, memory, and muscular contraction but it might also be detected in cells of non-neural origin, including fibroblast, immune cells, and cancer cells, where it is known to regulate immune functions, accelerate cell proliferation or support tumor dissemination [42].

The ability of BPDCN cells to exchange ACh was further supported by RNA sequencing validation results showing that, in BPDCNs versus normal pDCs, many genes belonging to the cholinergic machinery were significantly upregulated; among them, the nicotinic and muscarinic ACh receptor genes that are critically involved in cholinergic signaling activation and are under investigation for cancer therapy (Table S3). Muscarinic antagonists (e.g., darifenacin) are already in use for the treatment of genitourinary diseases, chronic obstructive pulmonary disease, and schizophrenia and have shown to arrest tumor growth in a small cell lung cancer mouse model [43]. Apart from genes, also two miRNA—miR-125b and miR-18, defined in literature as “CholinomiRs” [39]—were aberrantly upregulated in BPDCN samples, possibly contributing to the alteration of the ACh signaling at the post-transcriptional level.

In BPDCN, the cholinergic pathway might induce tumor growth akin to in lung cancer [44] and also promote cell migration by immune response modulation, as observed during viral infection [45].

According to RNA sequencing results, in addition to cholinergic-related genes, BPDCN cases, compared to normal pDCs, overexpressed many genes usually contributing to the survival, development and function of the nervous system (e.g., gene coding for nerve growth factors, *GABA* receptors, and neurotrophic tyrosine kinase receptors) (Table S7). In this regard, the upregulation of neurotrophic tyrosine kinase receptor genes in BPDCN certainly evokes clinical interest. Specifically, NRTK1/2/3 genes were overexpressed in BPDCN and the NRTK1 promoter was acetylated in both patients analyzed, leading us to hypothesize that NRTK1 expression could be epigenetically sustained. Recently, Renosi et al. documented the monoallelic deletion of NTRK genes in BPDCN. But the epigenetic control reported in the current study (i.e., acetylation of NRTK1 promoter) may overcome these deletions [46]. The neurotrophic receptors, known to promote tumor cell proliferation, differentiation, and survival, are emerging as promising therapeutic targets. A growing number of preclinical and clinical studies are investigating the NTRK inhibitors’ efficacy in hematological malignancies, above all AML [47]. But in the light of these findings, special attention should be paid to BPDCN too.

Collecting miRNA network, IHC and sequencing results, the final output we gained was the picture of a BPDCN tumor with a neural-oriented transcriptional program (Figure 5). The expression of well-known neural genes like *EDN3*, *NLGN4X*, *UCHL-1* and *DCX* may induce BPDCN metastasis. On the other hand, the activation of neural receptor genes (i.e., Ach receptors) may enable the cross-talk between BPDCN cells and central nervous system (CNS) and establish a tumor-supportive niche. About 30% of BPDCN cases relapse in the CNS, indicating that selected tumor cells resistant to chemotherapy may seed the brain and enter in close communication with neuronal cells [2,48]. The BPDCN case-series analyzed in this study perfectly reflects this rate of CNS relapse; 3/11 patients with available clinical information presented tumor dissemination in CNS (27.3%). A phenomenon occurring also in other hematological diseases, worth being better understood and explored.

We report here, for the first time, the expression in BPDCN cases of cholinergic-related genes and neural-related genes (*EDN3*, *NLGN4X*, *DCX* and *UCHL-1*) that warrant further investigation. Future studies are needed to assess their mechanistic role in BPDCN metastasis and evaluate their prognostic and or therapeutic relevance in the BPDCN clinical setting.

In conclusion, the analysis of BPDCN microRNA profiling leads to extending our knowledge of BPDCN pathobiology and to designing a new BPDCN cell model, equipped with neural traits that could facilitate the interaction with the nervous system. New potential neural metastatic inducers have been identified, and novel therapeutic approaches based on the use of anti-neurogenic agents are envisaged.

## Data Availability

The data presented in this study are openly available in GEO on accession numbers [GSE184293, GSE62014, GSE84471, GSE164939].

## Supplementary Materials

The following are available online at https://www.mdpi.com/article/10.3390/cancers13184680/s1, Figure S1: Workflow, Figure S2: Quantitative reverse transcription PCR (RT-qPCR), Figure S3: EDN3 and NLGN4X expression in AML and T-ALL, Figure S4: Immunostaining for neural markers in BPDCN, Table S1: Clinical characteristics of discovery set patients, Table S2: Immunohistochemical characteristics of discovery set patients, Table S3: Patients’ clinical features of immunohistochemistry validation set, Table S4: MiRNAs differentially expressed between BPDCN and pDC samples, Table S5: MiRNAs commonly deregulated in BPDCN and AML, Table S6: Genes and miRNAs interactions of BPDCN miRNA regulatory network, Table S7: Neural genes found upregulated in RNA sequencing validation sets.
