# Supplementary material for: Newly-Discovered Neural Features Expand the Pathobiological Knowledge of Blastic Plasmacytoid Dendritic Cell Neoplasm
Source: Cancers (Basel). 2021 Sep 18;13(18):4680. doi: 10.3390/cancers13184680 (PMC8469149; doi:10.3390/cancers13184680)
Supplement: Supplementary file 1 [file cancers-13-04680-s001.zip › cancers-1381918-supplementary.pdf]

Supplementary Material: Newly-Discovered Neural Features Expand the Pathobiological Knowledge of Blastic Plasmacytoid Dendritic Cell Neoplasm

Maria Rosaria Sapienza, Giuseppe Benvenuto , Manuela Ferracin , Saveria Mazzara , Fabio Fuligni, Claudio Tripodo, Beatrice Belmonte, Daniele Fanoni, Federica Melle, Giovanna Motta, Valentina Tabanelli, Jessica Consiglio, Vincenzo Mazzara, Marcello Del Corvo, Stefano Fiori, Alessandro Pileri, Gaetano Ivan Dellino, Lorenzo Cerroni, Fabio Facchetti, Emilio Berti, Elena Sabattini, Marco Paulli, Carlo Maria Croce and Stefano A. Pileri

RNA Extraction Discovery Set

RNA was extracted from FFPE and fresh/frozen samples of the discovery set with the RecoverAll Total Nucleic Acid Isolation Kit (Ambion, Life Technologies, Carlsbad, CA, USA) and Trizol (Invitrogen, Life Technologies, Carlsbad, CA, USA), respectively. RNA was quantified using ND-1000 spectrophotometer running software version 3.0.1 (NanoDrop Technologies Inc., Rockland, DE, USA).

MiRNA Expression Quantification by qPCR

To validate miRNA NanoString assays results, qRT-PCR was used (Real Time 7900HT, Life Technologies, Carlsbad, CA, USA). Specifically, miR-92a and miR-720 were studied in 3 pDCs and 4 BPDCNs, all belonging to the discovery set, except 2 external BPDCN samples. For each sample, 10 ng of total RNA were reverse transcribed using the TaqMan MicroRNA reverse transcription kit and gene-specific stem-loop primers for each miRNA, according to vendor’s instructions (Life Technologies, Carlsbad, CA, USA). Real-time PCR was performed using TaqMan probes specific for each miRNA and for RNU44, the latter being used as an endogenous control (Life Technologies, Carlsbad, CA, USA) and an Applied Biosystems 7900HT Real-Time PCR System according to the manufacturer’s standard protocol. Differences in miRNA expression were calculated by the $2^{-\Delta\Delta Ct}$.

Gene expression Profiling Analysis Discovery Set

Raw gene expression signals generated by the Whole Genome DASL HT assay were downloaded from GEO database (GSE62014) (7). The AVGSignal was used to measure the expression level of the raw gene signals downloaded from GEO database (GSE62014). R statistical environment (version 3.5.1) was applied to conduct a first filtering step by flag detection: probes with more than 40% of low-quality values were removed, while the remaining probes, mapping on the same gene annotation, were averaged, log transformed and quantile normalized. Differentially expressed coding elements between patients and controls were identified using the samr R package. Cutoffs for significant changes were a logFC > |2.5| and FDR < 0.05.

Gene Set Enrichment Analysis and Differential Analysis of RNA Sequencing Validation Sets

Functional enrichment analysis was performed using the Broad Institute Gene Set Enrichment Analysis (GSEA) software (21) and GO term assignments available in the Molecular Signatures Database (MSigDB) (https://www.gsea-msigdb.org/gsea/msigdb/collections.jsp#C5, accessed on 21 August 2020) (‘‘c5.all.v6.0.symbols.gmt’’).For each RNA sequencing validation set, a pre-ranked GSEA (Java command line version 2-2.2.4) on the GO_neurogenesis gene list (GO:0022008) available in MSigDB was used [1]. Differential analysis of RNA sequencing validation set 1 was conducted as previously reported [2]. Differential analysis of RNA sequencing validation set 2 was conducted by means of DeSeq [3], on normalized data provided in silico (GSE76147).

Table S1. Characteristics of discovery set patients

Sample	Sex	Age, y	Examined tissue	BM	PB	LN	CNS	Therapy	F.up	Mo
BPDCN_1	M	41	LN (FFPE)	NA	NA	(+)	NA	NA	AWD	53
BPDCN_2	M	80	LN (FFPE)	(+)	NA	(+)	NA	Prednisone + Vinblastine (6 cycles)	DOD	5
BPDCN_3	M	63	LN (FFPE)	(+)	NA	(+)	NA	Daunoblastina + ARA-C (3+7)	ADF	36
BPDCN_5	F	70	LN (FFPE)	NA	NA	(+)	(+), not at onset	NA	NA	NA
BPDCN_8	F	11	SK (FFPE)	NA	(-)	(+)	(-)	ARA-C like (6 cycles)	DOD	7
BPDCN_10	M	38	SK (FFPE)	(+)	NA	(-)	(+), not at onset	ARA-C	DOD	39
BPDCN_12	M	58	SK (FFPE)	NA	NA	NA	(-)	RT+CHOP like	DOD	10
BPDCN_13	M	30	SK (FFPE)	NA	NA	NA	NA	Hyper-CVAD	AWD	20
BPDCN_14	M	77	SK (FFPE)	NA	NA	NA	NA	Died before	DOD	1
BPDCN_16	M	14	SK (FFPE)	NA	NA	NA	NA	ICE+ ARACYTIN	AWD	11
BPDCN_17	M	69	SK (FFPE)	NA	NA	NA	(+), not at onset	Radiotherapy	DOD	42
BPDCN_18	M	89	SK (FFPE)	(+)	NA	(-)	NA	Died before	DOD	2
BPDCN_19	M	67	SK (FFPE)	(+)	NA	(-)	NA	Cyclophosphamide, Mitoxantrone and Prednisolone	DOD	2
BPDCN_20	F	66	SK (CRYO)	(+)	(+)	(-)	(-)	CHOP (6 cycles)	DOD	20
BPDCN_21	M	64	SK (CRYO)	(+)	(+)	(-)	(-)	IVA (6 cycles)	DOD	26
BPDCN_22	M	64	SK (CRYO)	(+)	(+)	(-)	(-)	Hyper-CVAD	DOD	23
BPDCN_23	M	19	SK (CRYO)	(-)	(-)	(-)	(-)	CHOP + MTX (5 cycles)	DOD	76
BPDCN_24	M	58	SK (CRYO)	(-)	(-)	(-)	(-)	Hyper-CVAD	ADF	28
BPDCN_25	M	62	SK (CRYO)	(+)	(+)	(+)	(-)	Hyper-CVAD	ADF	21

Abbreviations: BPDCN, blastic plasmacytoid dendritic cell neoplasm; y, years; BM, bone marrow; PB, pheripheral blood; LN, lymph-node; CNS, central nervous sytem; F.up, follow up; Mo, months M, male; F, female; LN, lymph node; SK, skin; NA, not available; Y, yes; ARA-C, cytarabine; RT, radiotherapy; CHOP, Cyclophosphamide, Doxorubicin, Vincristine,Prednisone; Hyper-CVAD, alternate cycles of hyper-fractionated Cyclophosphamide, Vincristine, Doxorubicin, Dexamethasone, and Methotrexate and Cytarabine; ICE, Ifosfamide, Carboplatin, Etoposide; IVA, Ifosfamide, Vincristine, Actinomycin D; CHOP+MTX, cyclical chemotherapy with high-dose methotrexate and CHOP; DOD, died of disease; AWD, alive with disease; ADF, alive disease free.

Table S2. Immunohistochemical characteristic of discovery of set patients.

Sample	CD3	CD4	CD30	CD34	CD56	CD68/PGM1	CD123	CD303/BDCA2	TCL1	MPO	TdT	CD20
BPDCN_1	(-)	(+/-)	nd	(-)	(+)	(-)	(+)	(+)	(+)	(-)	(+)	(-)
BPDCN_2	(-)	(+)	nd	(-)	(-)	(-)	(+)	nd	nd	(-)	exc	nd
BPDCN_3	(-)	(+)	nd	nd	(+)	nd	(+)	nd	(+)	nd	(+)	nd
BPDCN_5	(-)	(+/-)	exc	nd	(+)	(-)	nd	nd	nd	(-)	nd	(-)
BPDCN_8	(+-)	(+)	(-)	(-)	(+)	(+-)	(+)	(+)	na	(-)	(-)	(-)
BPDCN_10	(-)	(+)	nd	nd	(+)	(-)	(+)	(+)	(+)	(-)	(-)	(-)
BPDCN_12	(-)	(-)	nd	(-)	(+)	(+-) (dots)	(+)	nd	nd	(-)	(+)	(-)
BPDCN_13	(-)	(+)	nd	nd	(+)	(+-) (dots)	(+)	(+)	(+)	(-)	(+-)	(-)
BPDCN_14	(-)	(-)	nd	(-)	(+)	(+) (dots)	(+)	nd	nd	(-)	(-)	(-)
BPDCN_16	(-)	(+)	nd	nd	(+)	(-)	(+)	nd	(+)	(-)	nd	(-)
BPDCN_17	(-)	(+)	nd	nd	(-)	(+-) (dots)	(+)	(+)	(+)	(-)	(+)	(-)
BPDCN_18	(-)	(+)	nd	(-)	(+-)	(+-) (dots)	(+)	nd	nd	(-)	(+-)	(-)
BPDCN_19	(-)	(+)	nd	nd	(+)	(+-) (dots)	(+)	nd	(+)	(-)	(+-)	(-)
BPDCN_20	(-)	(+)	(-)	(-)	(+)	(+/-)	(+)	(+)	(+)	(-)	(+/-)	(-)
BPDCN_21	(-)	(+)	(-)	(-)	(+)	(+/-)	(+)	(+)	(+)	(-)	(+)	(-)
BPDCN_22	(-)	(+)	(-)	(-)	(+)	(-)	(+)	(+)	(+)	(-)	(+)	(-)
BPDCN_23	(-)	(+)	(-)	(-)	(+)	(+/-)	(+)	(+)	(+)	(-)	(-)	(-)
BPDCN_24	(-)	(+)	(-)	(-)	(+)	(-)	(+)	(+)	(+)	(-)	(-)	(-)
BPDCN_25	(-)	(+ -)	(-)	(-)	(+)	(-)	(+)	(+)	(+)	(-)	(-)	(-)

Abbreviations: BPDCN, blastic plasmacytoid dendritic cell neoplasm; (+), positive > 75% cells ; (+ -), positive 50-75% cells; (- +), positive 25-50%; (-), negative, no cell; nd, not done; exc, exceptional.

Immunohistochemistry Validation

Immunohistochemistry validation assays were performed in an independent set of 15 FFPE BPDCNs, whose clinical characteristics are summarized in Table S3. For immunostaining, tissue sections were dewaxed and rehydrated. The antigen unmasking technique was performed using Target Epitope Retrieval Solutions, pH6 and pH9 in thermostatic bath at 98°C for 30 minutes. Subsequently, the sections were brought to room temperature and washed in PBS. After neutralization of the endogenous peroxidase with 3% H_2O_2 and Fc blocking by a specific protein block (Novocastra, UK), the tissue samples were incubated with the following primary antibodies: Mouse Monoclonal Beta III Tubulin (Clone 2G10, 1:50 pH6, Santa Cruz SC-80005), Mouse Monoclonal NF-L (Clone 8A1, 1:50 pH6, Santa Cruz SC-20012), Mouse Monoclonal NF-H (Clone N52.1.7, 1:200 pH9, Novocastra NCL-NF200-N52), Mouse Monoclonal Tyrosine Hydroxylase TH (Clone F11, 1:50 pH6, Santa Cruz SC-25269), Rabbit Polyclonal Doublecortin DCX (1:1500 pH6, Abcam ab18723), Rabbit Polyclonal PGP9.5 Neuronal Marker (1:200 pH6, Abcam ab15503), and Rabbit Polyclonal VAChT (1:100 pH6, Abcam ab235201). Staining was revealed by polymer detection kit (Novocastra) and DAB (3,3'-Diaminobenzidine, Novocastra) substrate-chromogen. The slides were counterstained with Harris hematoxylin (Novocastra). All the sections were analyzed under a Zeiss AXIOScope.A1 optical microscope (Zeiss, Germany) and microphotographs were collected using a Zeiss Axiocam 503 Color digital camera using the Zen 2.0 imaging software.

Table S3. Patients' clinical features of immunohistochemistry validation set.

Samples	Sex	Age (years)	Sites					Therapy	F.up	Months
			SK	BM	PB	LN	OTHER			
BPDCN_IHC1	F	67	D	-	-	-	-	ALL-type	AWD	11
BPDCN_IHC2	F	84	D	-	-	-	-	mCHT	DOD	6
BPDCN_IHC3	M	57	D	-	-	+	-	SCT	DTR	60
BPDCN_IHC4	M	75	L	-	-	-	-	RT	DNR	22
BPDCN_IHC5	M	70	D	+	+	+	MALT	ALL-type	DOD	12
BPDCN_IHC6	M	29	Mu	-	-	-	PH	ALL-type	ADF	60
BPDCN_IHC7	M	83	D	-	-	-	-	TSEB	DOD	6
BPDCN_IHC8	F	49	L	-	-	-	-	RT/ALL type	DOD	27
BPDCN_IHC9	M	60	Mu	-	-	-	-	PUVA	DOD	12
BPDCN_IHC10	F	30	Mu	+	-	+	-	ALL-type	LOST	7
BPDCN_IHC11	M	78	D	-	-	+	-	TSEB	LOST	?
BPDCN_IHC12	M	69	D	+	+	-	-	mCHT	AWD	20
BPDCN_IHC13	M	71	Mu	+	+	+	-	HyperCVAD	AWD	20
BPDCN_IHC14	M	66	NA	NA	NA	NA	NA	NA	NA	NA
BPDCN_IHC15	F	80	NA	NA	NA	NA	NA	NA	NA	NA

Abbreviations: M, male; F, female; SK, skin; BM, bone marrow; PB, peripheral blood; LN, lymph node; Mu, multiple noncontiguous skin lesions; D, diffuse skin involvement; L, localized skin disease; MALT, mucosa-associated lymphoid tissue;PH, pharynge; ALL-type, acute lymphoblastic leukemia-like therapy, mCHT, monochemotherapy; SCT, allogeneic stem cell transplantation ;RT Radiotherapy; TSEB Total Skin Electron; PUVA, psoralen plus ultraviolet A light; Hyper CVAD, hyperfractionated cyclophosphamide, vincristine, doxorubicin, dexamethasone, alternating with high-dose methotrexate and cytarabine; NA, not available; AWD, alive with disease; ADF, alive disease free;DOD, dead of disease; DTR, death therapy-related; DNR natural death.

Table S4. MiRNAs differentially expressed between BPDCN and pDC samples (logFC > |1.5| and FDR < 0.05).

Gene ID	log Fold Change	q-Value(%)
hsa-miR-1274a	3.893568351	0
hsa-miR-1274b	3.624826024	0
hsa-miR-720	3.460952643	0
hsa-miR-181a+miR-181d	3.282978419	0
hsa-miR-1260	3.19488231	0
hsa-miR-205	3.064856689	0.235102791
hsa-miR-451	2.892001207	0.707352746
hsa-miR-145	2.842174313	0
hsa-miR-100	2.55293777	0
hsa-miR-630	2.530492957	0
hsa-miR-199a-5p	2.494799767	0
hsa-miR-200a	2.333285171	0
hsa-miR-143	2.250936551	0
hsa-miR-199b-5p	2.18030282	0
hsa-miR-1246	2.169878752	0
hsa-miR-128	2.104851238	0
hsa-miR-125b	2.072345929	0
hsa-miR-497	2.024441352	0
hsa-miR-744-ns	1.920240575	0
hsa-miR-523	1.8233666	0
hsa-miR-199a-3p+miR-199b-3p	1.789994417	0
hsa-miR-640	1.718470636	0
hsa-miR-1305	1.689740444	0
hsa-miR-1975	1.684858017	0
hsa-miR-34a	1.666396214	0
hsa-miR-1203	1.654329655	0
hsa-miR-122	1.644720123	0
hsa-miR-494-ns	1.596480077	0.707352746
hsa-miR-125a-5p	1.578339011	0
hsa-miR-1974	1.572751326	4.481849353
hsa-miR-376c	1.519626174	0
hsa-miR-362-3p	-3.769473998	0
hsa-miR-532-3p	-3.226533814	0
hsa-let-7f	-2.641032038	0
hsa-miR-660	-2.462179869	0
hsa-miR-374a	-2.241159681	0
hsa-miR-532-5p	-2.234242731	0
hsa-miR-142-3p	-2.180455119	0.235102791
hsa-miR-223-ns	-2.149580293	0
hsa-miR-362-5p	-2.04593968	0
hsa-miR-16-ns	-2.014859381	0
hsa-miR-503	-1.996007439	0
hsa-miR-340	-1.946405481	0
hsa-miR-363	-1.898261636	0
hsa-miR-15a	-1.815085786	0
hsa-miR-26a-ns	-1.798097631	0
hsa-miR-329	-1.721369702	0
hsa-miR-590-3p	-1.65996248	0
hsa-miR-492	-1.641200431	0
hsa-miR-501-3p	-1.597176869	0
hsa-miR-142-5p-ns	-1.53522011	0.423674822

Table S5. miRNAs commonly deregulated in BPDCN and AML.

miRNAs	Up/Down	MiRNet	miRCancer	References
hsa-mir-100	Up	x		[4]
hsa-mir-34a	Up	x		[5]
hsa-mir-1246	Up	x		[6]
hsa-mir-128	Up	x	x	[7]
hsa-mir-125b	Up	x	x	[8]
hsa-mir-181a	Up	x	x	[9]
hsa-mir-340	Down		x	[10]
hsa-miR-142-3p	Down		x	[11]
hsa-miR-223	Down		x	[12]

Table S6. Gene and miRNA interactions of BPDCN miRNA regulatory network.

miRNA	Interaction	gene name
hsa-miR-604	interacts with	ZC3H18
hsa-miR-604	interacts with	CFHR4
hsa-miR-604	interacts with	FAM107A
hsa-miR-604	interacts with	CRTC1
hsa-miR-639	interacts with	HIST2H3A
hsa-miR-639	interacts with	CDKN1A
hsa-miR-639	interacts with	HIST2H3C
hsa-miR-639	interacts with	DR1
hsa-miR-639	interacts with	NLGN4X
hsa-miR-640	interacts with	ENPP6
hsa-miR-640	interacts with	CYP4X1
hsa-miR-342-5p	interacts with	XIRP1
hsa-miR-342-5p	interacts with	FAM107A
hsa-miR-342-5p	interacts with	OSBPL10
hsa-miR-342-5p	interacts with	ASB6
hsa-miR-342-5p	interacts with	LRCH1
hsa-miR-342-5p	interacts with	CRTC1
hsa-miR-342-5p	interacts with	ARSE
hsa-miR-342-5p	interacts with	PAAF1
hsa-miR-532-5p	interacts with	ABCA13
hsa-miR-532-5p	interacts with	TRAPPC2B
hsa-miR-532-5p	interacts with	SEPTIN14
hsa-miR-532-5p	interacts with	NMUR2
hsa-miR-532-5p	interacts with	RAP2A
hsa-miR-484	interacts with	NUDT17
hsa-miR-484	interacts with	BLOC1S3
hsa-miR-484	interacts with	ASB6
hsa-miR-484	interacts with	PITPNA
hsa-miR-484	interacts with	NMT2
hsa-miR-501-3p	interacts with	FOS
hsa-miR-1275	interacts with	MAT1A
hsa-miR-1275	interacts with	GNG2
hsa-miR-1275	interacts with	FTO
hsa-miR-1275	interacts with	CRTC1
hsa-miR-1275	interacts with	TSKU
hsa-miR-1275	interacts with	GLI4
hsa-miR-1275	interacts with	FAM102A
hsa-miR-1275	interacts with	PITPNA

hsa-miR-1275	interacts with	PRKAG3
hsa-miR-1275	interacts with	POU3F1
hsa-miR-1275	interacts with	TMEM104
hsa-miR-1275	interacts with	PELI2
hsa-miR-1275	interacts with	TTC31
hsa-miR-1275	interacts with	SOX10
hsa-miR-1275	interacts with	SCRT1
hsa-miR-1275	interacts with	ZMIZ2
hsa-miR-1275	interacts with	ORMDL3
hsa-miR-492	interacts with	AHCY
hsa-miR-492	interacts with	TBC1D9B
hsa-miR-492	interacts with	TSKU
hsa-miR-492	interacts with	ORMDL3
hsa-miR-663b	interacts with	CRTC1
hsa-miR-663b	interacts with	TSKU
hsa-miR-663b	interacts with	PYCR1
hsa-miR-663b	interacts with	ORMDL3
hsa-miR-532-3p	interacts with	OMP
hsa-miR-532-3p	interacts with	CYP4X1
hsa-miR-532-3p	interacts with	TNNC2
hsa-miR-508-3p	interacts with	GSTA2
hsa-miR-508-3p	interacts with	DIAPH2
hsa-miR-508-3p	interacts with	GRIA4
hsa-miR-508-3p	interacts with	RAP2A
hsa-miR-1246	interacts with	HTN1
hsa-miR-1246	interacts with	OGN
hsa-miR-875-3p	interacts with	TMEM139
hsa-miR-875-3p	interacts with	MAP1B
hsa-miR-875-3p	interacts with	PELI2
hsa-miR-875-3p	interacts with	DOLPP1
hsa-miR-875-3p	interacts with	STAMBPL1
hsa-miR-1203	interacts with	EDN3
hsa-miR-1203	interacts with	TMEM104
hsa-miR-1253	interacts with	DIAPH2
hsa-miR-1253	interacts with	FOS
hsa-miR-1253	interacts with	NMUR2
hsa-miR-1253	interacts with	PYCR1

Table S7. Neural genes found upregulated in RNA sequencing validation sets.

**RNA sequencing Validation set 1**		
**Gene**	**logFC**	**adj.P.Val**
ACHE	4.461281	5.67E-10
BCHE	4.742431	0.00039
CHRM1	5.56408	0.000251
CHRM2	4.477625	0.036287
CHRNA3	4.038435	0.000199
CHRNA4	4.550008	0.03677
CHRNA5	3.491177	1.06E-06
CHRNA6	7.204784	4.33E-05
CHRNB1	1.303299	0.002751
CHRNB3	5.206778	0.011014
CHRNB4	5.25403	0.008532
GABARAP	1.141662	0.029202
GABARAPL3	4.70697	0.026526
GABBR2	3.947962	0.042128
GABRA2	4.613405	0.030132
GABRA3	4.980122	0.015195
GABRA4	9.319402	1.31E-08
GABRB1	6.481679	0.000668
GABRB2	1.768269	0.053744
GABRD	2.310871	0.023102
GABRE	6.585138	3.52E-07
GABRG1	5.278046	0.010996
GABRG2	4.765532	0.026036
GABRG3	4.523235	0.034409
GABRP	7.461487	2.83E-05
GABRQ	4.077755	0.017324
GABRR1	4.116145	0.009394
NGF	5.977137	0.002163
NGFR	2.652573	0.000135
NTRK1	3.236966	5.88E-05
NTRK2	9.885109	2.25E-21
NTRK3	5.808321	2.39E-06
RNA sequencing Validation set 2		
**Gene**	**logFC**	**adj.P.Val**
GABBR1	2.994333	5.34E-05
GABPB2	1.885167	0.002786
GABRR2	3.264667	0.014338

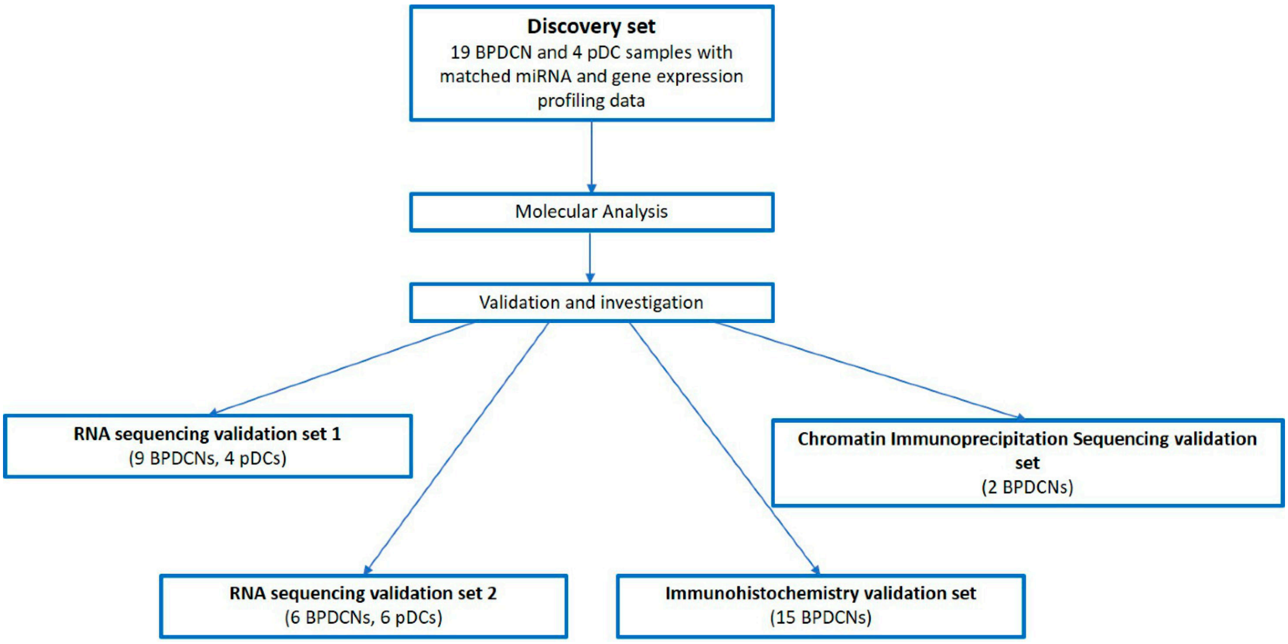

Figure S1. Workflow**.** The study design has been graphically summarized. Molecular analysis was conducted on the discovery set and the resulting molecular findings were next validated and explored in RNAsequencing validation sets 1 and 2, immunohistochemistry and H3K27me3/ac Chromatin Immunoprecipitation Sequencing validation sets.

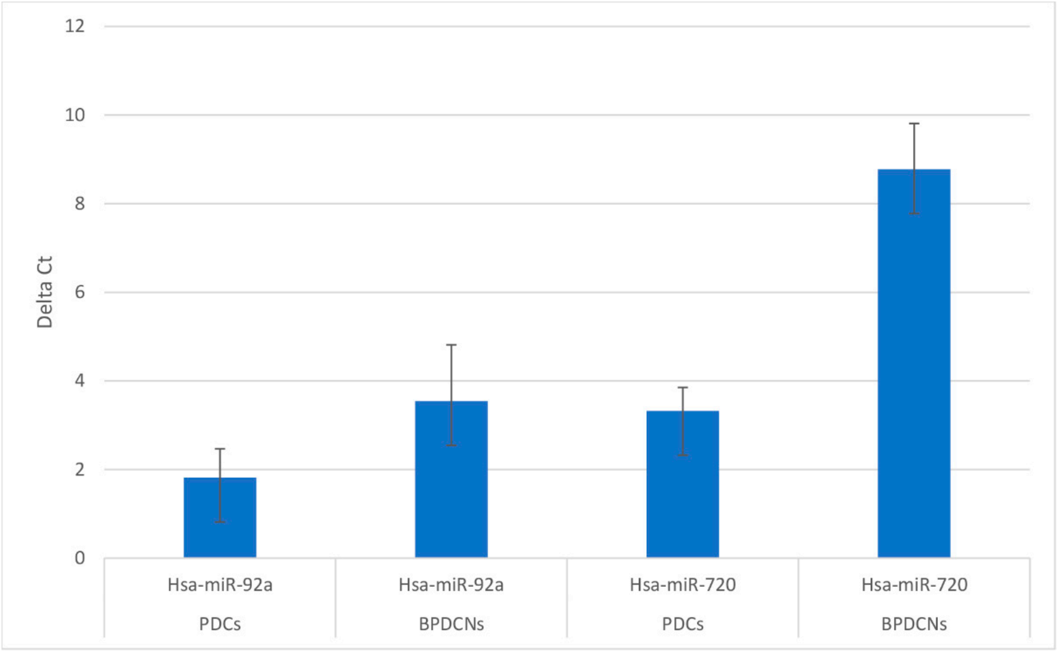

Figure S2. Quantitative reverse transcription PCR (RT-qPCR)**.** Histogram represented the fold change expression value of miR-92a and miR-720 in BPDCNs respect to pDCs.

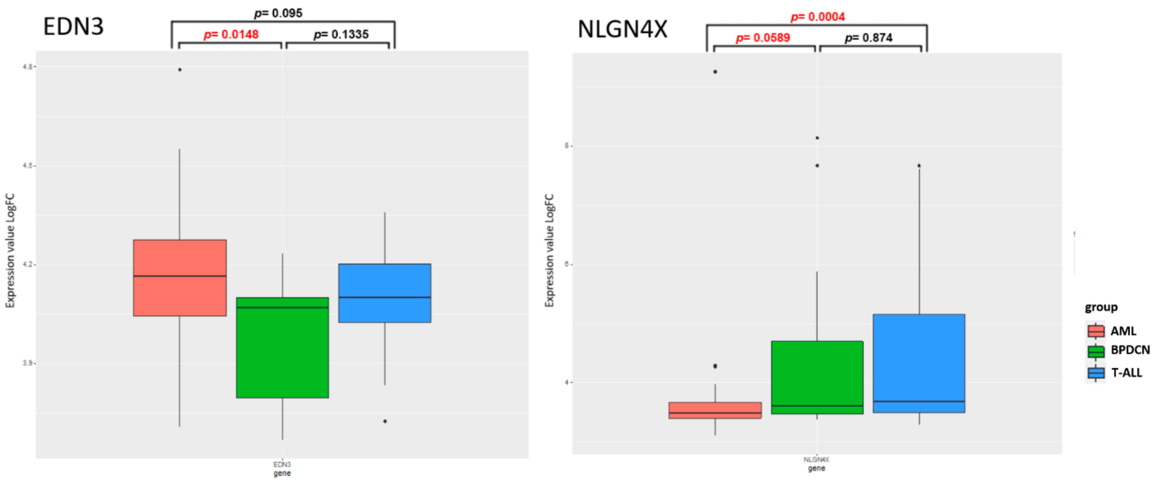

Figure S3. EDN3 and NLGN4X expression in BPDCN, AML and T-ALL. Box plots show on the left the expression of EDN3 gene and on the right the expression of NLGN4X gene in 65 AMLs (red boxes), 12 BPDCNs (green boxes) and 35 T-ALLs (blue boxes), respectively. The gene expression data was downloaded from GEO database (GSE89565)[13]. *EDN3* was significantly upregulated in AML vs BPDCN and *NLGN4X* was significantly upregulated in BPDCN vs AML and in T-ALL vs AML (Mann Whitney test *p*-value ≤ 0.05).

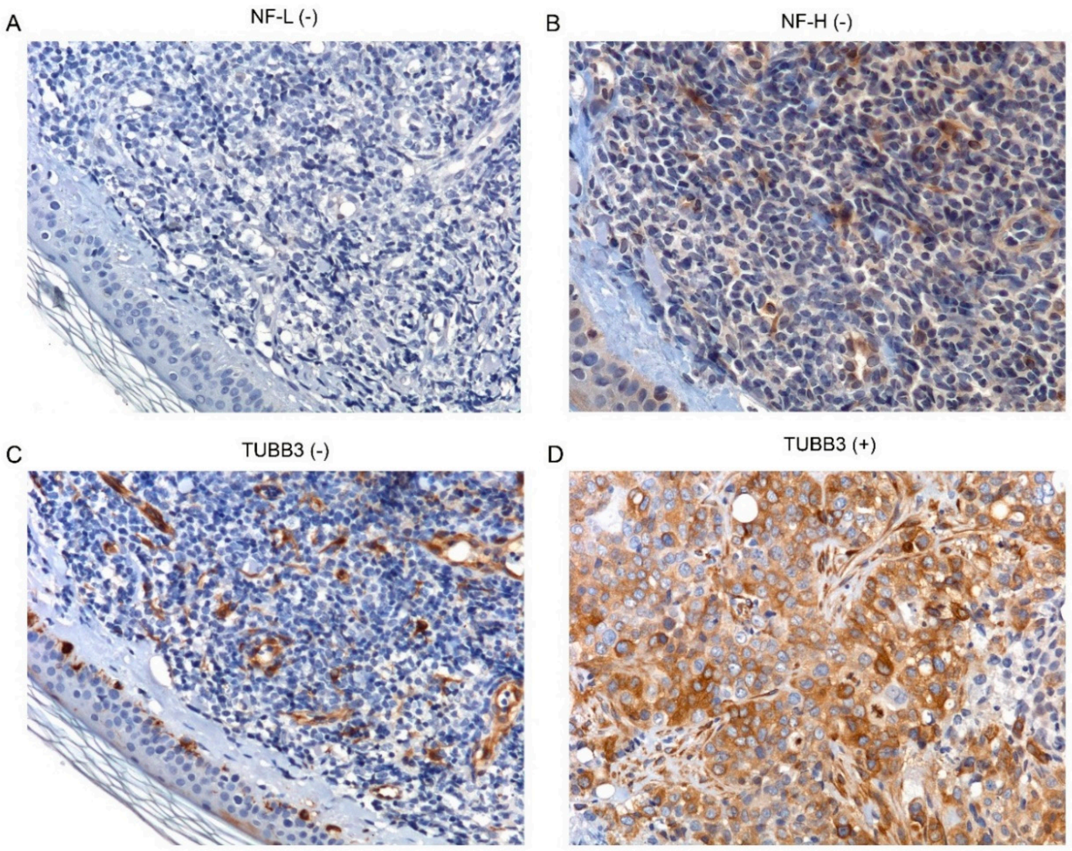

Figure S4. Immunostaining for neural markers in BPDCN. Immunohistochemistry assay reported the negativity of BPDCN tumor cells for NF-L (**A**) NF-H (**B**). (**C**) Fourteen out of fifteen BPDCN cases presented tumor cells negative for TUBB3 while surrounding melanocytes and endothelial cells were positive for TUBB3. (**D**) only one BPDCN sample has tumor cells positive for TUBB3. Original magnifications × 20.

References

1. Liberzon, A.; Subramanian, A.; Pinchback, R.; Thorvaldsdóttir, H.; Tamayo, P.; Mesirov, J.P. Molecular Signatures Database (MSigDB) 3.0. *Bioinformatics* **2011**, *27*, 1739–1740, doi:10.1093/bioinformatics/btr260.
2. Sapienza, M.R.; Abate, F.; Melle, F.; Orecchioni, S.; Fuligni, F.; Etebari, M.; Tabanelli, V.; Laginestra, M.A.; Pileri, A.; Motta, G.; et al. Blastic Plasmacytoid Dendritic Cell Neoplasm: Genomics Mark Epigenetic Dysregulation as a Primary Therapeutic Target. *Haematologica* **2019**, *104*, 729–737, doi:10.3324/haematol.2018.202093.
3. Anders, S.; Huber, W. Differential Expression Analysis for Sequence Count Data. *Genome. Biol.* **2010**, *11*, R106, doi:10.1186/gb-2010-11-10-r106.
4. Zheng, Y.-S.; Zhang, H.; Zhang, X.-J.; Feng, D.-D.; Luo, X.-Q.; Zeng, C.-W.; Lin, K.-Y.; Zhou, H.; Qu, L.-H.; Zhang, P.; et al. MiR-100 Regulates Cell Differentiation and Survival by Targeting RBSP3, a Phosphatase-like Tumor Suppressor in Acute Myeloid Leukemia. *Oncogene* **2012**, *31*, 80–92, doi:10.1038/onc.2011.208.
5. Isken, F.; Steffen, B.; Merk, S.; Dugas, M.; Markus, B.; Tidow, N.; Zühlsdorf, M.; Illmer, T.; Thiede, C.; Berdel, W.E.; et al. Identification of Acute Myeloid Leukaemia Associated MicroRNA Expression Patterns. *Br. J. Haematol.* **2008**, *140*, 153–161, doi:10.1111/j.1365-2141.2007.06915.x.
6. Chen, L.; Guo, Z.; Zhou, Y.; Ni, J.; Zhu, J.; Fan, X.; Chen, X.; Liu, Y.; Li, Z.; Zhou, H. MicroRNA-1246-Containing Extracellular Vesicles from Acute Myeloid Leukemia Cells Promote the Survival of Leukemia Stem Cells via the LRIG1-Meditated STAT3 Pathway. *Aging (Albany NY)* **2021**, *13*, 13644–13662, doi:10.18632/aging.202893.
7. Seca, H.; Lima, R.T.; Almeida, G.M.; Sobrinho-Simoes, M.; Bergantim, R.; Guimaraes, J.E.; Vasconcelos, M.H. Effect of MiR-128 in DNA Damage of HL-60 Acute Myeloid Leukemia Cells. *Curr. Pharm. Biotechnol.* **2014**, *15*, 492–502, doi:10.2174/1389201015666140519122524.
8. Liu, J.; Guo, B.; Chen, Z.; Wang, N.; Iacovino, M.; Cheng, J.; Roden, C.; Pan, W.; Khan, S.; Chen, S.; et al. MiR-125b Promotes MLL-AF9-Driven Murine Acute Myeloid Leukemia Involving a VEGFA-Mediated Non-Cell-Intrinsic Mechanism. *Blood* **2017**, *129*, 1491–1502, doi:10.1182/blood-2016-06-721027.
9. Huang, X.; Schwind, S.; Santhanam, R.; Eisfeld, A.-K.; Chiang, C.-L.; Lankenau, M.; Yu, B.; Hoellerbauer, P.; Jin, Y.; Tarighat, S.S.; et al. Targeting the RAS/MAPK Pathway with MiR-181a in Acute Myeloid Leukemia. *Oncotarget* **2016**, *7*, 59273–59286, doi:10.18632/oncotarget.11150.
10. Wang, Q.; Feng, T.; Xu, J.; Miao, M.-H.; Ji, X.-Q.; Zhu, H.; Shao, X.-J. Low Expression of MicroRNA-340 Confers Adverse Clinical Outcome in Patients with Acute Myeloid Leukemia. *J. Cell. Physiol.* **2019**, *234*, 4200–4205, doi:10.1002/jcp.27178.
11. Zhang, Y.; Liu, Y.; Xu, X. Upregulation of MiR-142-3p Improves Drug Sensitivity of Acute Myelogenous Leukemia through Reducing P-Glycoprotein and Repressing Autophagy by Targeting HMGB1. *Transl. Oncol.* **2017**, *10*, 410–418, doi:10.1016/j.tranon.2017.03.003.
12. Wallace, J.A.; O'Connell, R.M. MicroRNAs and Acute Myeloid Leukemia: Therapeutic Implications and Emerging Concepts. *Blood* **2017**, *130*, 1290–1301, doi:10.1182/blood-2016-10-697698.
13. Ceroi, A.; Masson, D.; Roggy, A.; Roumier, C.; Chagué, C.; Gauthier, T.; Philippe, L.; Lamarthée, B.; Angelot-Delettre, F.; Bonnefoy, F.; et al. LXR Agonist Treatment of Blastic Plasmacytoid Dendritic Cell Neoplasm Restores Cholesterol Efflux and Triggers Apoptosis. *Blood* **2016**, *128*, 2694–2707, doi:10.1182/blood-2016-06-724807.
